# Kaposi’s Sarcoma-Associated Herpesvirus ORF57 Protein: Exploiting All Stages of Viral mRNA Processing

**DOI:** 10.3390/v5081901

**Published:** 2013-07-26

**Authors:** Sophie Schumann, Brian R. Jackson, Belinda Baquero-Perez, Adrian Whitehouse

**Affiliations:** School of Molecular and Cellular Biology, and Astbury Centre for Structural Molecular Biology, University of Leeds, Leeds LS2 9JT, UK

**Keywords:** KSHV, herpesvirus, ORF57, TREX, mRNA export, mRNA stability

## Abstract

Nuclear mRNA export is a highly complex and regulated process in cells. Cellular transcripts must undergo successful maturation processes, including splicing, 5'-, and 3'-end processing, which are essential for assembly of an export competent ribonucleoprotein particle. Many viruses replicate in the nucleus of the host cell and require cellular mRNA export factors to efficiently export viral transcripts. However, some viral mRNAs undergo aberrant mRNA processing, thus prompting the viruses to express their own specific mRNA export proteins to facilitate efficient export of viral transcripts and allowing translation in the cytoplasm. This review will focus on the Kaposi’s sarcoma-associated herpesvirus ORF57 protein, a multifunctional protein involved in all stages of viral mRNA processing and that is essential for virus replication. Using the example of ORF57, we will describe cellular bulk mRNA export pathways and highlight their distinct features, before exploring how the virus has evolved to exploit these mechanisms.

## 1. Introduction

Kaposi’s sarcoma-associated herpesvirus (KSHV) or human herpesvirus 8 (HHV-8), belongs to the lymphotropic (γ) herpesvirus subfamily [1] and is the causative agent of three human cancers: multicentric Castleman’s disease (MCD) [2], primary effusion lymphoma (PEL) [3], and Kaposi’s sarcoma (KS) [4]. While PEL and MCD are both rare B-cell lymphoproliferative disorders, KS, an aggressive tumour of endothelial origin, has become one of the most common cancers in many sub-Saharan African countries where individuals are co-infected with HIV and KSHV [5]. Moreover, KS develops in states of immunosuppression and additionally affects patients after organ transplantation [6]. KS is characterised by an abnormal neovascularisation which thrives in a rich inflammatory microenvironment, initially affecting the skin and later progressing to internal organs and lymph nodes [7]. 

Similar to other herpesviruses, KSHV can follow a latent or lytic replication programme in the infected host. The virus life cycle starts with replication in its primary target, B-cells from peripheral blood [8] and the oropharynx [9], and also in oral epithelial cells, which enables transmission between individuals by saliva [10,11]. The virus establishes latency in the majority of these infected cells, during which the genome is maintained as a large circular episome in the nucleus of the cell and only a very small subset of viral genes are expressed, resulting in no infectious virions being released. Under certain stimuli, such as hypoxia [12], lytic replication is reactivated in a small proportion of cells leading to expression of more than 80 transcripts, release of infectious virions and cell lysis. Importantly, lytic replication allows dissemination of the virus from the initial reservoir of infected cells to endothelial cells where tumours may develop. In the KS tumour, infected endothelial cells adopt characteristic spindle morphology with most remaining latently infected and few undergoing lytic replication [13]. Unlike other human oncogenic herpesviruses, such as Epstein-Barr (EBV) virus, in which the latent phase plays a key role in tumorigenesis [14], both the latent and the lytic phase are required for KS tumour development, with several lytic proteins exhibiting putative tumorigenic activities [15]. Hence, deciphering the molecular mechanisms that allow for lytic replication to occur are of great interest, as this may lead to a better understanding of KSHV-mediated pathogenesis and present novel approaches for therapeutic drugs targeting this process*.*

This review will focus on the role of KSHV ORF57 (also called MTA-mRNA transcript accumulation) in the lytic life cycle of the virus. ORF57 is essential for virus lytic replication and functions in all stages of viral mRNA processing with a particular role in viral mRNA export. To highlight the role of ORF57 the concept of mammalian mRNA export will first be explored.

## 2. Cellular mRNA Export and Viruses

### 2.1. Introduction to Cellular mRNA Export

A characteristic feature of eukaryotic cells is compartmentalization, which necessitates the presence of specific transport mechanisms allowing macromolecules to travel between the compartments. One such group of macromolecules are cellular mRNAs, which are produced in the nucleus, but must be exported into the cytoplasm in order for translation to occur. The physical separation of mRNA production and utilisation allows for the presence of multiple regulatory and quality control mechanisms. Accordingly, the majority of primary transcripts undergo multiple specific maturation steps turning precursor mRNA (pre-mRNA) to mRNA, including splicing, 5'-, and 3'-end processing. Failure to proceed through this process results in nuclear retention or degradation of the mRNA [16,17,18,19,20,21]. Opposed to this, successful mRNA processing is directly linked to nuclear export, as each step triggers recruitment of protein factors necessary for mRNA export [22,23,24].

Viruses replicating their genome in the nucleus of the host cell also face the challenge of specifically exporting their mRNAs into the cytoplasm. In this section we will highlight cellular mRNA export pathways and show how some viruses exploit or circumvent these pathways for their own replication, before exploring KSHV ORF57-mediated mRNA export in detail.

### 2.2. Cellular Bulk mRNA Export and the TREX Complex

When leaving the nucleus, all mRNA needs to pass through selective and well controlled channels in the nuclear envelope: the nuclear pore complexes (NPCs), formed by nucleoporins. Hence, all export-competent ribonucleoproteins (RNPs) contain specific adapter proteins and export receptors that are targeted to the NPC. Cellular bulk mRNA export is facilitated via the export adapter transcription-coupled export (TREX) complex and the export receptor Nxf1/TAP (Mex67 in *S. cerevisiae*) and Nxt1/p15 (Mtr2 in *S. cerevisiae*) [25,26,27]. Table 1 lists all known components of TREX. 

**Table 1:** Known components of the human transcription-coupled export (TREX) complex.

TREX Component	Alternative Name	*S. cerevisiae* Ortholog	Known Interactions in TREX
**UAP56**	BAT1, DDX39B	Sub2	Aly, CIP29 [28], Chtop [29], UIF [30], THO [23], SKAR, ZC11A [31]
**DDX39**	URH49, DDX39A	Sub2	Aly [32], UIF [30], CIP29 [33]
**Aly**	Ref, Alyref, Thoc4, Bef	Yra1	UAP56 [34], Chtop [29], Thoc5, Thoc2 [35]
**CIP29**	HCC1, Tho1, Sarnp	Tho1	UAP56 [28]
**UIF**	FYTTD1	-	UAP56 [30]
**Chtop**	SRAG, CAO77 FOP	-	UAP56, Aly [29]
**SKAR**	pDIP3, PolDIP3	-	?
**ZC11A**	ZC3H11A	-	?
**Thoc1**	Hpr1, p84	Hpr1	Part of THO [35]
**Thoc2**	Tho2	Tho2	Aly, Part of THO [35]
**Thoc5**	fSAP79, Fmip	-	Aly, Part of THO [35]
**Thoc6**	fSAP35, WDR58	-	Part of THO [35]
**Thoc7**	fSAP24	-	Part of THO [35]
**Tex1**	Thoc3	Tex1	Part of THO [35]

**Figure 1:**
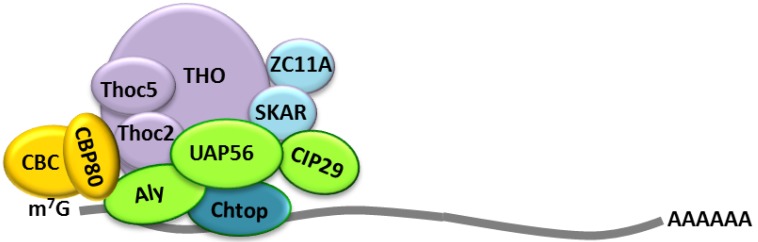
Formation of transcription-coupled export (TREX) on the 5'-end of the mRNA through direct interactions with the cap-binding complex (yellow).

Studies using various model organisms have shown a great degree of conservation of the TREX complex between plants, yeast and higher eukaryotes [25,36,37,38]. In this review we will focus on the human TREX complex (hereafter referred to as TREX), unless otherwise specified. Figure 1 provides some schematics showing known components and assembly of the TREX complex.

Since mRNAs differ greatly, recognition of cargo by the TREX complex must be independent of features such as sequence, length and structure. Exceptions are found in less abundant types of mRNA, such as intron-less mRNAs, where only recently a specific consensus element was identified to recruit the TREX complex [39,40]. Instead, nuclear bulk mRNA export is directly linked to mRNA biogenesis, coupling successful mRNA processing and maturation to a stepwise assembly of the mRNP. The first processing event is the addition of a 5' cap, where a 7-methylguanosine (m^7^G) is added by an unusual 5'-5' linkage to the first nucleotide of the transcript, in order to protect the nascent pre-mRNA from 5'–3' degradation [41]. Nuclear export is regulated by the 5' cap, as localisation of the TREX complex to the 5' end of the mRNA has been shown to be cap dependent. Aly and THO, two components of the TREX complex, interact directly with cap binding protein 80 (CBP80), a member of the cap-binding complex (CBC) [22,35,42] (Figure 1). Consequently, uncapped mRNAs have been shown to be poorly exported from the nucleus in microinjected *Xenopus* oozytes [22]. 

**Figure 2:**
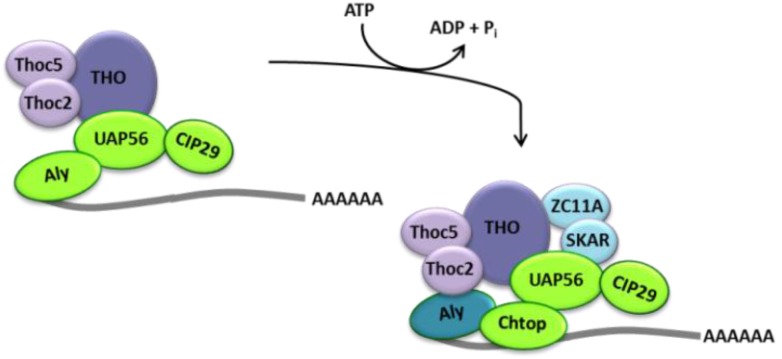
UAP56 assembles the TREX complex in an ATP-dependent manner.

After capping, the transcript is spliced to remove the non-coding intronic regions. In 2000 and 2001, studies emerged linking splicing to mRNA export in higher eukaryotes through a direct interaction of the export adapter protein Aly and a conserved DEAD-box helicase UAP56 [24,43]. Subsequent studies found Aly and UAP56 interact with a protein complex known as the exon-junction complex (EJC), which is formed 20–24 nucleotides upstream of each exon-exon junction [44]. Because of its association with export factors, the EJC was initially suggested to form a link between splicing and export, but more recently evidence emerged showing that the EJC functions in processes distinct to TREX and post-mRNA export, such as enhancement of translation, mRNA localization and nonsense-mediated decay (NMD) [45,46,47]. Two specific proteins in the EJC have been associated with translational enhancement of spliced transcripts, namely PYM and SKAR [48,49]. However, an independent study also identified SKAR as a component of the TREX complex [28]. Furthermore, SKAR, together with the protein ZC11A, has been shown to associate with TREX components in an ATP-dependent manner and play a role in mRNA export [31]. This suggests that the components of each complex may be multifunctional and there may also be dynamic remodeling and interplay between the EJC and TREX, involving individual members of both complexes. Further work, therefore, is needed to clarify the role of SKAR and whether the protein, as initially suspected with UAP56 and Aly, might be part of both the EJC and TREX and therefore provide a link between these two complexes. Both Aly and UAP56 have since been shown to be central components of the TREX complex, where they form an ATP-dependent trimeric complex with CIP29 [28]. Furthermore, the UAP56 paralogue, DDX39, which shares 90% sequence identity and 96% sequence similarity, has also been shown to interact with Aly [32] and CIP29 [33]. While UAP56 and DDX39 have redundant and overlapping functions in TREX, DDX39 has been proposed to target a functionally distinct subset of mRNAs [33]. UAP56 also associates with Chtop, a protein first identified by proteomic analysis of immunopurified TREX [28], in an ATP-dependent manner [29]. While CIP29 can bind UAP56 simultaneously with Aly, Chtop binding can be outcompeted by the presence of Aly, indicating consecutive loading of both proteins onto TREX. Recruited by UAP56, which acts as an ATP-dependent assembly factor, these TREX components bind exclusively to the 5'-end of the mRNA (Figure 2) [22,28,29]. Hence, both capping and splicing are necessary for assembly and function of the TREX complex [22,23,24]. 

TREX formation in yeast appears to be dependent on transcription. During this process the THO complex—tightly associated proteins forming a sub-complex of the TREX complex—associates with the nascent RNA. It is believed that in yeast the THO complex recruits the remaining TREX complex. Accordingly, yeast strains with dysfunctional THO subunits show a severe blockage at mRNA export [50]. In contrast, knockdown of metazoan THO subunits results only in limited loss of function, with the vast majority of mRNAs still being exported [51]. Previous reports show that THO associates directly with the RNA-helicase UAP56, but unlike the interactions of UAP56 with Aly and CIP29, this interaction takes place in an ATP-independent manner [28]. In contrast, a more recent study suggests that THO associates only with Aly and not UAP56 [35]. Results show a direct interaction between Aly and the THO components Thoc2 and Thoc5, contradicting previous findings [28,52] and highlighting the complexity of working with a dynamically remodeled multi-protein complex. While knockdown of UAP56 and its paralogue, DDX39, abolishes virtually all mRNA export, redundancy has been reported for other components of TREX, for example Aly. Serine/arginine rich proteins (SR proteins) 9G8, SRp20 and SF2/ASF can link export receptor Nxf1/TAP-binding to spliced mRNA [53]. Furthermore, UIF (UAP56 interacting factor) has been shown to interact with UAP56 and Nxf1/TAP [30]. Importantly, individual knockdown of Aly or UIF does not significantly inhibit bulk mRNA export, however, depletion of both proteins leads to an almost complete loss of mRNA export, showing a similar phenotype as UAP56/DDX39 knockdown. This adds to a further complexity of the key components of TREX with redundant proteins having essential roles in mRNA export. 

It is also believed that the final mRNA maturation steps, 3'-end cleavage and polyadenylation, are coupled to the recruitment of Aly onto the TREX complex. In yeast, the Aly homologue Yra1 is recruited to the UAP56 homologue Sub2 via the 3'-end processing factor Pcf11. Sub2 uses the same binding region in Yra1 that is necessary for the interaction with Pcf11, suggesting mutually exclusive interactions [54]. Furthermore, it has been shown that Pcf11 displacement by an RNA and ATP loaded Sub2 gives way for Clp1, a subunit of the cleavage-polyadenylation factor CF1A, to bind Pcf11 [55]. Since this interaction is essential for a functioning cleavage-polyadenylation complex, successful mRNA 3'-end processing is coupled to formation of the TREX complex [56,57]. The interaction of Aly and Pcf11 is conserved in metazoans, also indicating coupling of mRNA 3'-end processing and export in higher organisms [54]. Interestingly, a recent report indicates Aly is not the only TREX component with an influence on 3'-end processing [58]. Thoc5 has been found to interact with pre-mRNA cleavage and polyadenylation factor CFIm68 and recruit it onto the 5'-end of target genes. This interaction then affects a choice of alternative polyadenylation sites and lengthening of the 3'-UTR. Considering that alternative polyadenylation is known to be a control mechanism for gene expression [59], this highlights new roles of THO in TREX and pre-mRNA processing.

**Figure 3:**
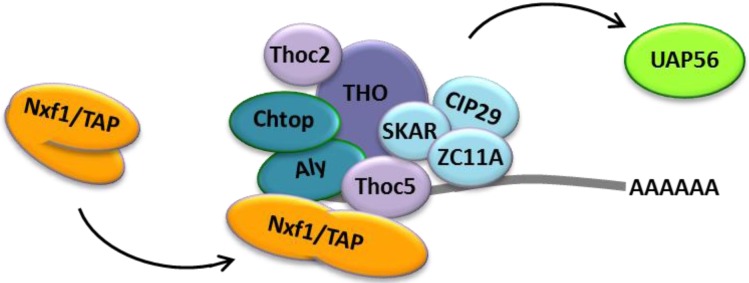
Binding of Nxf1/TAP to Aly and Thoc5 induces a conformational change in the protein which allows RNA binding [60] and leads to the release of UAP56.

As mentioned previously, an mRNP requires the additional recruitment of export receptors that specifically interact with the NPC to be considered export competent. For this, TREX acts as a binding platform for the non-karyopherin heterodimer of Nxf1/TAP and Nxt1/p15. Once ATP-bound UAP56 is associated with Aly, CIP29, Chtop and the THO complex, Aly and Thoc5 bind Nxf1/TAP directly [52]. This induces a conformational change in the export receptor which allows for RNA binding [60]. Furthermore, binding and handover of mRNA to Nxf1/TAP leads to displacement of UAP56 which then leaves TREX [61], as shown in Figure 3. 

**Figure 4:**
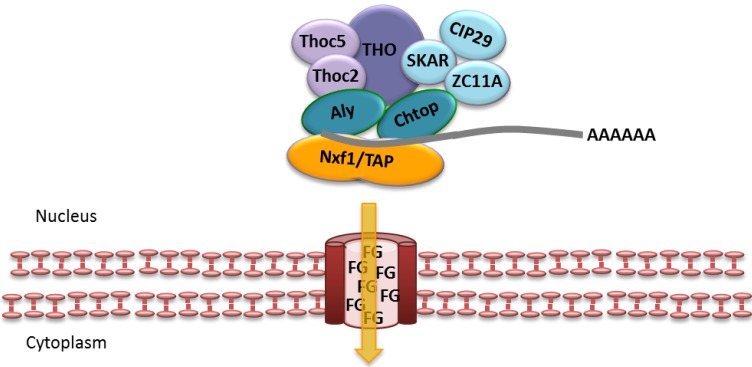
Handover of mRNA from TREX onto Nxf1/TAP and translocation through the nuclear pore complex (NPC).

Post-translational methylation of Aly within its Nxf1/TAP- and RNA-binding domain reduces its affinity for RNA and facilitates the handover of the mRNA from Aly to Nxf1/TAP [61,62]. Recent research has also shown that Chtop is able to bind Nxf1/TAP, a process necessary for mRNA handover from Aly to Nxf1/TAP [29]. The interaction of Chtop with Nxf1/TAP, which is regulated by the methylation status of Chtop, is mutually exclusive of Thoc5, however, both proteins exist in the same complex [29]. This indicates further remodeling of the complex and as such the order of binding and remodeling at the point in mRNA export remains to be fully elucidated (Figure 4). However, the mRNA transfer allows docking of the mRNP to the NPC via a transient interaction of Nxf1/TAP and Nxt1/p15 with phenylalanine/glycine (FG)-rich nucleoporins and subsequent translocation through the central channel (for a more detailed review see [63]). 

Several human herpesviruses, including KSHV, Epstein-Barr virus, herpes simplex virus type 1 and human cytomegalovirus, have been shown to recruit various components of the TREX complex onto their mRNA in order to facilitate nuclear export of viral transcripts. As this forms the focus of our review, we will explore this area in further detail below. Simple retroviruses have also been shown to utilise human Nxf1/TAP and Nxt1/p15 to facilitate nuclear export of specific viral pre-mRNAs, but without association of the TREX complex as an adapter protein complex. Interestingly, research into the nuclear export of the incompletely spliced mRNA from Mason Pfizer Monkey Virus (MPMV) first identified Nxf1/TAP as a nuclear export factor in metazoans and gave way to research uncovering the major cellular mRNA export pathway [64,65]. The incompletely spliced RNAs from MPMV were found to contain a highly structured, *cis*-acting sequence, termed the constitutive transport element (CTE), sufficient for nuclear export [64]. Studies using type D retrovirus RNAs subsequently identified Nxf1/TAP as the cellular factor directly binding to the CTE and facilitating nuclear export [65]. Influenza A virus has also been shown to utilise the Nxf1/TAP pathway [66]. Interestingly, different subsets of viral mRNA appear to have different requirements for the presence of TREX components and/or Nxf1/TAP. While viral intron-less mRNAs coding for early gene products showed a weak dependency on Nxf1/TAP, export of late intron-less mRNA was strongly dependent on the presence of the nuclear export receptor. Furthermore, export of unspliced, intron containing viral mRNAs was reliant on both Nxf1/TAP and UAP56, whereas Aly, in addition to UAP56 and Nxf1/TAP was also recruited to spliced viral mRNA. This indicates that spliced viral mRNAs are processed in a manner similar to cellular transcripts and recruit the complete TREX complex, whereas unspliced orintron-less mRNAs utilise a viral adapter protein for the interaction with UAP56 and/or Nxf1/TAP. However, this adapter protein has yet to be identified. 

### 2.3. CRM1-Dependent mRNA Export

While the CRM1-dependent export pathway is commonly used to export non-coding RNAs, such as ribosomal RNAs (rRNAs) and small nuclear RNAs (snRNAs) [67], as well as proteins [68], only a very small subset of cellular mRNAs utilise this pathway. Prominent examples are *Cd83*, *Fos* and *cyclin D1* [69,70,71]. CRM1 (also known as exportin-1) belongs to the conserved karyopherin family of nuclear export receptors. It is structurally different from Nxf1/TAP, lacks RNA binding properties, but recognises leucine-rich-type nuclear export signals (NES) within proteins [72,73]. Accordingly, an NES containing adapter protein is needed for bridging the mRNA–CRM1 interaction. All karyopherins are regulated by the small GTPase Ran [74,75]. Exportins, such as CRM1, only bind their cargo in the nucleus when loaded with RanGTP and dissociate from it upon GTP hydrolysis. A gradient of RanGTP across the nuclear membrane generates the driving force and ensures directionality of transport towards the cytoplasm, where only GDP-bound Ran exists [76]. The presence of RanGAP (Ran-GTPase-activating protein) in the cytoplasm ensures GTP hydrolysis and release of the cargo from CRM1 [76]. 

The CRM1 dependent export pathway is exploited by complex retroviruses, such as HIV, for exporting their unspliced or partially spliced mRNAs. HIV, like all members of the lentivirus family, expresses Rev as a viral adapter protein. Rev contains a leucine-rich sequence serving as an NES and is therefore able to bind CRM1 [73]. It also binds RNAs containing a highly structured *cis-*acting motif termed the Rev response element (RRE), which enables Rev to bridge the interaction between RNA and export receptor [77,78]. Another well characterised viral interaction partner for CRM1 is Rex, expressed by human T-cell leukemia virus (HTLV), which functions similar to Rev [79,80]. A recent review by Shida gives further details about RNA export in retroviruses [81].

### 2.4. Nuclear Budding of Large Nuclear mRNPs

Recently it has been reported for the first time that some mRNAs leave the nucleus without traveling through the NPC. This novel pathway describes large mRNPs leaving the nucleus by budding from the inner and outer nuclear membrane in *Drosophila* muscle cells [82]. RNA granules were found to associate with *C*-terminal fragments of a Wg receptor involved in Wnt-signalling during synapse development. The exported transcripts encoded postsynaptic proteins, which function locally in synapse assembly [82]. Mutations interfering with foci assembly caused formation of undifferentiated synapse boutons, which provides a fascinating connection to previous studies describing diseases such as muscular dystrophies as a result of faulty proteins of the inner-nuclear membrane [82,83,84]. Moreover, the mechanism of this process is beginning to be dissected, as the AAA-ATPase torsin has been identified as a key component of this pathway [85]. In mutants deficient in torsin, these large mRNPs are not exported and instead accumulate in the perinuclear space. Interestingly, this pathway bears great similarity to herpesvirus capsids budding from the nuclear membrane after assembly in the nucleus. These multimegadalton complexes are too large to travel through the NPC and instead leave the nucleus by inner-nuclear-membrane envelopment and outer-nuclear-membrane de-envelopment [86]. 

## 3. ORF57

Research over the last decade from several laboratories has identified KSHV open reading frame (ORF) 57 that functions as a viral SR protein with multiple roles throughout viral mRNA processing [87,88,89,90,91], like its well studied homologues in other herpesviruses: ICP27 of herpes simplex virus type 1 [92,93], Mta/SM of Epstein-Barr virus [94,95], UL69 of human cytomegalovirus [96], ORF4 of varicella-zoster virus [97], and ORF57 of herpesvirus saimiri [98,99,100,101]. The multiple functions of KSHV ORF57 range from potential roles in transcription and splicing to viral mRNA stability and export, as well as translational enhancement [87,91,102,103,104,105]. Due to this far-reaching multi-functionality, ORF57 is able to interact with multiple viral and cellular proteins. The following section will explore the role of ORF57 in virus lytic replication. 

### 3.1. ORF57 Interactions with TREX to Mediate Export of Viral mRNA

Herpesviruses, including KSHV, replicate in the nucleus of the host cell and produce numerous lytic intron-less mRNAs. However, as previously mentioned, the processes of transcription, splicing and mRNA nuclear export are intimately linked. Indeed, the recruitment of protein complexes including TREX and the EJC are splicing-dependent [23]. This poses a significant stumbling block for KSHV lytic replication, as numerous viral mRNAs are intron-less, meaning they do not undergo splicing and therefore cannot recruit TREX via the splicing-dependent mechanism. To overcome this obstacle and promote efficient nuclear export of viral lytic intron-less transcripts, KSHV encodes the immediate-early protein ORF57 [106,107]. 

**Figure 5:**
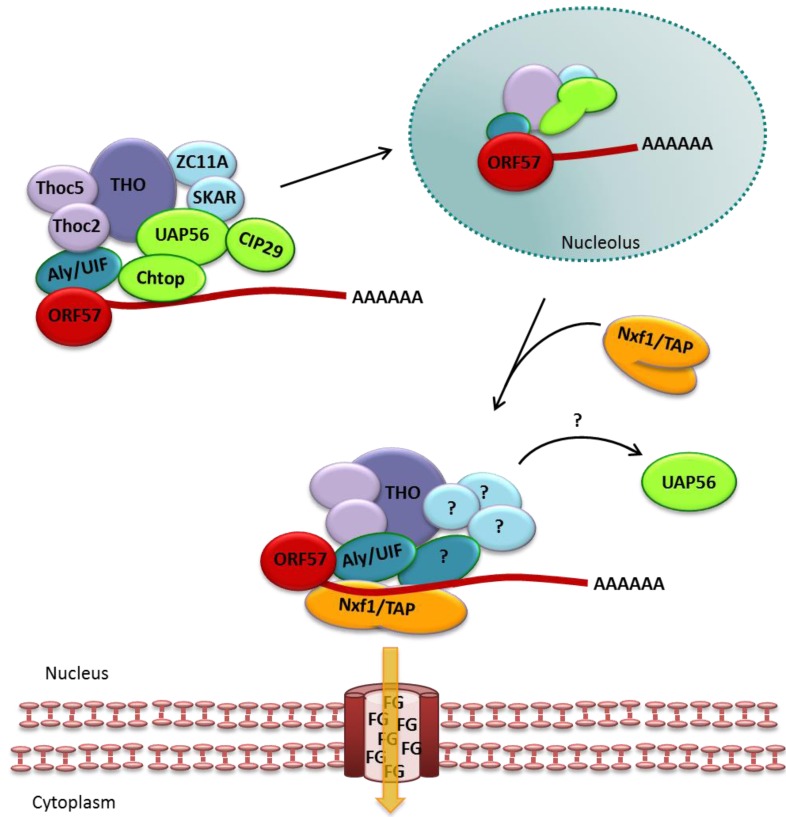
Open reading frame (ORF) 57 recruits the TREX complex onto viral mRNA, relocalises to the nucleolus and leaves the nucleus through the NPCs.

The first interaction identified between ORF57 and a TREX complex component was with the export adapter protein Aly [90]. ORF57 is now known to bind directly to Aly and through this interaction recruit UAP56 and subsequently the entire TREX complex onto viral mRNA (Figure 5). Similar to mammalian mRNA export mechanisms, Aly bridges the interaction to Nxf1/TAP which facilitates viral mRNA export through the NPC [87]. Notably, trans-dominant Aly mutants which prevented assembly of the complete TREX complex, but were still able to interact with ORF57 as well as Nxf1/TAP, showed a decrease in viral intron-less mRNA export and virus replication [87]. Hence, the presence of ORF57, Aly and the nuclear export factor Nxf1/TAP on viral mRNA alone are not sufficient for vRNP export. Another interesting observation is that ORF57 shuttles through the nucleolus during the viral mRNA export process. Accordingly, all components of TREX can be seen to relocalise to the nucleolus with ORF57 and, importantly, the nucleolus appears to be essential for mRNA export both in KSHV [88] and in the model gamma-2 herpesvirus, herpesvirus saimiri [98]. However, the exact reason for this relocalisation remains to be elucidated, although it is clear that shuttling throughout different cellular compartments is an essential feature of ORF57. Current evidence suggests that shuttling between the nucleus and nucleolus occurs prior to exiting the nucleus into the cytoplasm and then being recycled into the nucleus again [107] (Figure 5). 

This process is aided by three nuclear localization signals (NLS) encoded within ORF57, each of which is sufficient to function as an independent NLS and with the first two NLS functioning together as a nucleolar localization signal [98,107,108]. At which point ORF57 interacts with each of its protein partners throughout this shuttling process is currently not fully clear, and neither is the exact mechanism of nuclear import. Heterokaryon assays demonstrate the shuttling of ORF57 between the nucleus and cytoplasm [109] and work on herpesvirus saimiri suggests that ORF57 homologues can interact with members of the importin family [110,111], although to date no direct interaction has been shown between KSHV ORF57 and importins. Clearly, better understanding of the exact shuttling mechanisms of ORF57 is essential to fully understand its functional role within KSHV lytic replication.

Interestingly, some reports have suggested that the ORF57-Aly interaction is not required for either mRNA export or viral replication [112,113]. Surprisingly, siRNA knockdown of Aly does not completely prevent viral mRNA export [105]. However, an explanation for this observation can be seen in the inherent redundancy within cellular mRNA export. Knock down of Aly in a cellular system results in an increase of a second compensatory export adapter, UIF, meaning that mRNA export can still occur, albeit at a slightly lower level [30]. Importantly, ORF57 has also been shown to interact directly with UIF and through this same redundancy mechanism recruit both Aly and UIF to viral mRNA to facilitate mRNA export [105]. Furthermore, siRNA knockdown of both Aly and UIF not only results in dramatically lowered levels of mRNA export, but also in a decrease of viral protein expression. This highlights the importance of the ORF57-TREX interaction mediated by Aly and UIF. Moreover, although ORF57 binds directly with either Aly or UIF to recruit TREX, it also interacts with other cellular proteins involved in mRNA export. Interaction with the cellular mRNA export factors OTT3 and RBM15 has been shown to be important for the expression of KSHV transcripts [114]. A similar scenario was believed to be the case for herpes simplex virus type 1, where the interaction between Aly and the ORF57 homologue ICP27 was thought to be dispensable for mRNA export, however, recent analysis has shown for the first time that this interaction is required for efficient export of viral mRNA [115]. Mutational analysis based on previous structural data [116] demonstrated that knocking out three key residues in the ICP27-Aly interaction inhibited mRNA export, despite the fact that ICP27 has been shown to directly interact with TAP/Nxf1 [117].

ORF57 homologues have also been shown to enhance viral mRNA export through interaction with TREX. The IE4 protein of varicella-zoster virus interacts with several cellular export factors including ASF/SF2, 9G8, SRp20, Aly and TAP, and has been shown to facilitate the export of viral mRNAs via the TAP/Nxf1 export pathway [97]. Also, the Epstein-Barr virus EB2 protein interacts directly with Aly to promote viral mRNA export [118]. Interestingly, a direct interaction with Aly is not essential for all herpesviruses to export their mRNA, for example, human cytomegalovirus UL69 directly interacts with the DEAD-box helicase UAP56 [119], an essential component of TREX. These findings suggest that while the mechanism of interacting with the human cellular export machinery may vary between herpesviruses, the requirement of recruiting TREX to viral mRNAs via ORF57 and its homologues appears to be evolutionarily conserved.

### 3.2. ORF57 Functions Additional to Viral mRNA Export

#### 3.2.1. ORF57 Acts to Stabilise Viral mRNA

It has been known for some time that as well as functioning in viral mRNA export, ORF57 also acts to stabilise RNA [113,120]. How ORF57 is recruited to viral RNAs has long been investigated, and it was not until recently that the first work identifying a mechanism was published. The majority of work investigating the enhancement of RNA stability has been performed on a KSHV non-coding RNA termed PAN (polyadenylated nuclear RNA), a long non-coding RNA (lncRNA), which contains a recognition sequence for ORF57, or ORE (ORF57 response element). The PAN ORE is a nine nucleotide sequence confirmed by two independent studies and is able to confer ORF57 responsiveness when transferred to a reporter construct [121,122]. Interestingly, a further ORE has also been identified in the mRNA encoding vIL-6 consisting of the four core bases of the PAN ORE [123]. In both situations the ORE allows ORF57 binding and subsequent enhancement of RNA stability, although the mechanism by which stability is increased may differ. ORF57 has been shown to bind directly to the cellular polyadenylate-binding protein cytoplasmic 1 (PABPC1) and that PABPC1 is necessary for ORF57 to bind to the PAN ORE [124]. ORF57 also relocalises the majority of PABPC1 to the nucleus where PAN is retained. These observations suggest that PABPC1 is important for the stabilization of PAN in an ORF57-dependent manner. The mechanism of RNA stability of vIL-6 is slightly better understood. Binding of ORF57 to the mRNA via this minimal ORE acts as a competitive binding mechanism and protects the mRNA from miRNA-mediated degradation. Interestingly, ORF57 acts in a similar way to protect the cellular IL-6 mRNA from degradation, suggesting that ORF57 stabilises both viral and cellular mRNAs [123]. There are, however, several important questions remaining regarding ORF57-mediated RNA stabilization. As mentioned, work in this area is limited primarily to the non-coding PAN RNA, and a more in depth analysis of other viral, as well as cellular, RNAs would help to understand the role of ORF57 in RNA stability. It is also interesting to note that whilst PAN is the most highly expressed lytic transcript the function of PAN remains largely unknown. Moreover, ORF57 has been shown to stabilise several KSHV mRNAs (including ORF47, ORF59 and PAN) but have no effect on others (GCR and K5) [87,125], so understanding which RNAs ORF57 interacts with, the mechanism by which it is recruited to viral RNAs, and for what reason—whether it be stability, export or both—remain important questions.

#### 3.2.2. Transcriptional Enhancement by ORF57

ORF57 function is not limited to interactions with cellular proteins or at the post-transcriptional level, it has also been shown to form protein-protein interactions with the viral protein RTA (replication transcriptional activator). Besides ORF57, RTA is the second protein essential for virus lytic replication, since it functions as a viral switch to induce lytic replication in latently infected cells [126,127]. By binding directly to promoter regions containing an RTA responsive element (RRE), RTA transactivates several viral and cellular promoters [128]. It is believed that ORF57 interacts with RTA to enhance the expression cascade of viral proteins and thereby increase the efficiency of KSHV reactivation. While ORF57 contains an A/T hook domain in the *N*-terminal region that confers DNA binding and has been shown to function as a transactivator for some viral genes, the role of ORF57 as a transcriptional enhancer in lytic replication seems to be dependent on the interaction with RTA [103,125,129]. The interaction with ORF57 has been found to enhance the effect of RTA on several promoters, such as PAN/nut-1, Kaposin, *ori-Lyt* (L), K-bZIP, and TK [103,125,129]. It has to be noted though, that the synergistic effect of ORF57-RTA is promoter-, transcript-, and cell line-specific [129]. More recently, ORF57 has also been shown to bind directly to the KSHV transcription factor K-bZIP [130]. ChIP analysis demonstrated that ORF57 associates with the viral genome, with particular enrichment at the promoters of ORF4 and K-bZIP, in regions where K-bZIP itself binds. This further highlights the role ORF57 plays to regulate viral gene expression.

#### 3.2.3. The Role of ORF57 in Translational Enhancement

Cellular mRNA processing is not only necessary for assembly of TREX, but also leads to the deposition of the EJC on the mRNA. As mentioned previously, the EJC is important for nonsense-mediated decay, mRNA localization and importantly translational enhancement [45,46,47]. SKAR and PYM, protein members of the EJC, have been shown to interact with multiple other translational enhancement proteins, resulting in enhancement of the pioneer round of translation [48,49]. PYM specifically is able to recruit the 48S pre-initiation complex through a direct interaction with the small ribosomal subunit [48].

**Figure 6:**
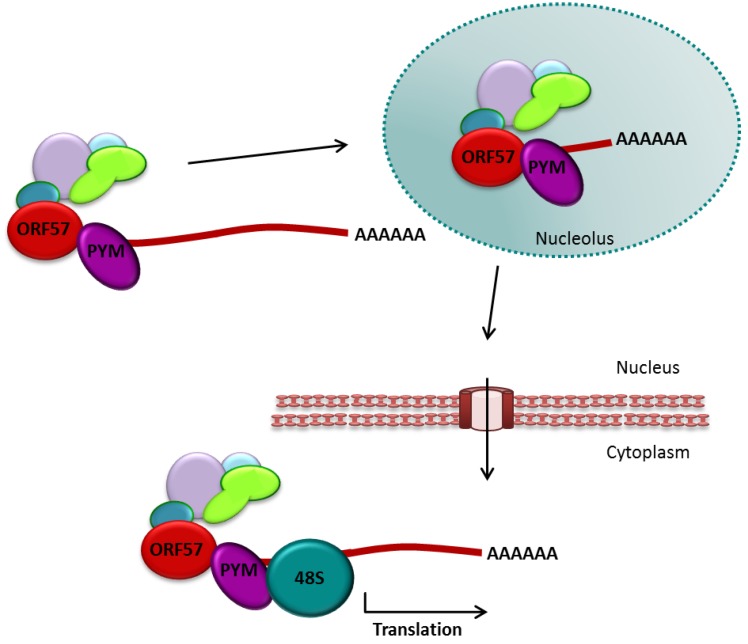
ORF57 interacts directly with PYM to enhance translation.

Since the EJC is deposited on the mRNA in a splicing dependent manner, the virus is facing a conundrum similar to mRNA export. In order to circumvent this splicing-dependent step, ORF57 interacts directly with PYM, which is sufficient to facilitate the assembly of the 48S pre-initiation complex onto viral intron-less mRNA (Figure 6) [104,131]. 

Presence of a PYM mutant, which does not interact with either the EJC or 48S pre-initiation complex, but retained ORF57 binding ability, drastically reduced the presence of late KSHV proteins as well as KSHV virion production. Interestingly, ORF57 failed to interact with or recruit other components of the EJC onto viral intron-less transcripts [104]. Together these results highlight the role of the PYM-ORF57 interaction in translational enhancement. Intriguingly, while the role of ORF57 in intron-less mRNA export is conserved amongst herpesviruses, no other ORF57 homologue has been shown to interact with PYM to enhance translation to date.

#### 3.2.4. ORF57 and Splicing of Viral Transcripts

While numerous KSHV genes are intron-less, the virus also encodes multiple spliced transcripts. It is therefore not surprising that the virus should aim to enhance splicing of these genes. Accordingly, ORF57 has been shown to form a complex with the spliceosome and enhance splicing of viral lytic genes [91]. Specifically, the protein is able to enhance splicing of constructs containing a large exon upstream of a small intron, including viral and non-viral pre-mRNA constructs. This exon-intron distribution is found in the majority of KSHV spliced genes, but is distinct to mammalian mRNAs with small exons and large introns. Accordingly, constructs with oversized exons are only poorly spliced by the cellular splicing machinery, warranting ORF57 interference. While ORF57 has been found interacting with pre-mRNA in the presence of nuclear extracts, this could not be seen in the presence of purified ORF57, indicating that the interaction is not direct, but facilitated by cellular proteins, and that ORF57 utilises those proteins to modulate normal splicing [91]. A similar effect has been seen by ORF57 EBV homolog SM, which acts to influence the choice of splice site [132].

## 4. Concluding Remarks

Since the discovery of KSHV in 1994 [4] there has been a large body of work investigating the latent-lytic switch and the immediate-early proteins that regulate the lytic cycle. The role that ORF57 plays in all aspects of viral gene expression is critical to the full completion of the lytic cycle, as seen in ORF57 knock-out recombinant KSHV, which does not produce infectious virions [133]. Moreover, ORF57 homologues are conserved across all herpesviruses, their common function being to enhance viral transcript export and accumulation, highlighting the importance of this protein in the herpesvirus life-cycle.

Our knowledge of the mechanisms surrounding nuclear mRNA export is still in its infancy. As such, our understanding of how viral export factors interact with these dynamic protein complexes and facilitate viral mRNA export is limited by our understanding of the cellular mechanisms. The recent discovery that ATP-driven remodeling of TREX takes place to allow mRNA export is then complicated by the addition of viral factors. ORF57 is known to bind directly to both Aly and UIF to recruit the complete TREX complex, but the mechanism by which TREX is then recruited is not fully understood. It is possible that the addition of ORF57 to this complex has no effect on the ATP-driven remodeling and handover of mRNA from Aly to TAP/Nxf1 and that ORF57 simply acts as a bridge between the viral mRNA and human export pathway. However, it would be interesting to investigate whether ORF57 interacts with TREX in such a way as to subvert the normal mechanism of mRNA export in any way, particularly as ORF57 is able to utilise either Aly or UIF for recruitment of TREX and export of viral mRNA [87,105].

One important aspect of KSHV ORF57-mediated viral mRNA export that should be investigated further is the binding domains within ORF57 and Aly/UIF. Two recent studies highlighted the essential residues for binding of the ORF57 homologues ICP27 of herpes simplex virus, and ORF57 of herpesvirus saimiri to Aly [115,116]. Interestingly, removal of these binding sites severely hampered efficient mRNA export despite the previous observation in KSHV that UIF provides mRNA adapter redundancy. To date, KSHV ORF57 is the only herpesvirus mRNA export factor that has been shown to interact with UIF, however, there is no published work for or against the interaction with other herpesvirus ORF57 homologues. Interestingly, competition assays showed that ORF57 may preferentially bind Aly over UIF but this could also suggest that Aly and UIF share a binding site on ORF57 [105]. Furthermore, siRNA depletion of Aly and UIF separately has a moderate effect on mRNA export compared to near complete abolishment of export in a double knockout suggesting that both Aly and UIF could be required for the efficient recruitment of TREX for viral mRNA export.

The high incidence of KS in the developing world means that the need for novel KSHV therapeutics is as essential as ever [5,134]. Importantly, knocking out ORF57 function can prevent the full lytic cycle due to the essential nature of viral mRNA export, making ORF57 an ideal target for novel therapeutics. Furthermore, studying the mechanism of mRNA export by ORF57 could help to further our understanding of various aspects of mRNA export. Needless to say, the study of ORF57 and its homologues in viral mRNA export has far-reaching implications in both our understanding of cellular mechanisms and in potential new anti-viral and anti-cancer therapeutics. 
